# Placental transport of Erythromycin and its effect on placental inflammatory factors

**DOI:** 10.12669/pjms.39.1.6683

**Published:** 2023

**Authors:** Hassan Elbiss, Nawal Osman

**Affiliations:** 1 Hassan M Elbiss, Departments of Obstetrics and Gynaecology, College of Medicine and Health Sciences, UAE University, Al Ain, UAE; 2 Nawal Osman, Departments of Obstetrics and Gynaecology, College of Medicine and Health Sciences, UAE University, Al Ain, UAE

**Keywords:** Erythromycin, Placenta, Maternal-Fetal Exchange

## Abstract

**Objective:**

Erythromycin is used for prevention and control of infectious perinatal morbidity. It has been hypothesised that erythromycin crosses the placenta and has an effect on the production of placental inflammatory factors. We evaluated the transport of erythromycin in an ex-vivo closed perfusion system of the placenta and determined its effect on the production of placenta inflammatory markers.

**Methods:**

In 2013, a prospective basic science study was conducted at the placental laboratory of College of Medicine and Health Sciences, United Arab Emirates. Six term placentas from uncomplicated pregnancies were studied using the ex-vivo dual closed-loop human placental cotyledon perfusion technique. Erythromycin was added to the perfusate in the maternal compartment. Samples were obtained from the maternal and fetal up to 240 minutes.

**Results:**

The reference antipyrine was detected in the fetal circulation in the first 15 minutes after addition of the drug. At this point the mean antipyrine was 49.90±2.10μg/ml in the maternal perfusate and 7.1±1.56μg/ml in fetal perfusate. The fetal and maternal concentration became similar at 120 minutes. The transfer of antipyrine from maternal to fetal compartment was 98.66%. The differences between perfusion groups were non-significant that indicates the perfusion of placentas was comparable. After media exchange in both sides, erythromycin was added to the maternal perfusate. The experimental period of four hours was continued with medium circulation on both maternal and fetal circulation. The concentration of erythromycin decreased in the maternal circuit by 36.4% and increased in the fetal circuit by 65%. The concentration of IL-6 in the maternal circuit was normal.

**Conclusion:**

Erythromycin crossed the placenta and did not inhibit the production of IL-6. Future studies are needed concerning neonatal adverse effects and the development of antibiotic resistance.

## INTRODUCTION

The exposure of women during pregnancy to drugs and environmental contaminants is considered as high risk. As shown in our other study^1^ establishment of a maternal-fetal vascular interface, a feature vital for successful pregnancy outcome,^1^ involves remodeling of the maternal spiral arterioles in the myometrium and decidua by the endovascular trophoblasts early in pregnancy. The placenta plays a role in drug transfer contributing to infectious disease vertical transmission and prevention. Preterm birth, a major public health challenge related to disabilities in the offspring, is associated in 40% of cases with preterm prelabour rupture of membranes (PPROM).^2^ Intrauterine infection has been associated with preterm babies with cerebral palsy and chronic lung disease.^3^

Antibiotics, e.g. erythromycin, have been used prophylactically in women with PPROM associated with significant reductions in chorioamnionitis, numbers of babies born within 48 hours and seven days, neonatal infection and other related outcomes in the newborn.^4^ Inflammatory factors play a role in offspring outcome as bronchoalveolar lavage fluid from infants who developed chronic lung disease contains a higher concentration of profibrotic agent transforming growth factor B.^5^ Antibiotics, including erythromycin, have also been recommended if Group-B Streptococcus (GBS) is found in a vaginal swab as an incidental finding or in women with a previous baby affected by neonatal GBS disease.^6^ Thus, it can be hypothesised that erythromycin crosses the placenta and has an effect on the production of placental inflammatory factors.

The objective of our work was to determine the transport of erythromycin in an ex-vivo closed perfusion system of the placenta and to determine the effect of erythromycin on the production of inflammatory markers produced by the placenta.

## METHODS

We conducted a prospective basic science study of three months duration in 2013 at the placental laboratory of the College of Medicine and Health Sciences, United Arab Emirates.

### Placental collection:

Six placentas were obtained from healthy women with uncomplicated term pregnancies, undergoing vaginal delivery at Tawam Hospital, Al Ain, United Arab Emirates. The mothers all provided informed consent, and the study protocol was approved by the ethical committee of College of Medicine and Health Science, United Arab Emirates (Ethics board approval number is AAMDHREC10/61 and registered date is 01/06/2012). The human placenta perfusion method is well recognized to deploy a sample size of six placentas.

### In vitro perfusion method:

The perfusion technique has been described in detail by Schneider and Pangel^7^ After delivery the placentas, with cords clamped, were immediately transported to perfusion laboratory, a suitable normal looking cotyledon with intact decidual plate was selected and distal branches from chorionic artery and vein were cannulated. The cotyledon was cut from the placenta and placed in the perfusion chamber with maternal side upwards. Four needles were placed 2-3mm below maternal surface in intervillous space. The cannulated fetal side was also fixed to the chamber. The maternal outflow, coming from the decidual plate, was drained outside the perfusion chamber. The perfusion was started with flow rate of 4ml/minute on fetal side and 12ml/minute on maternal side. Media are continuously gassed with atmospheric air (95% air and 5% carbon dioxide) on the maternal side, and 95% nitrogen and 5% carbon dioxide on the fetal side throughout the experiment. The perfusate in each side was composed of tissue culture medium 199 with Earl’s salts, sodium bicarbonate, heparin (2500 IU/L), and dextran. The drug tested, Erythromycin, was obtained from Sigma Aldrich Chemicals, CO, St Louis, Missouri, USA.

### Perfusion experiment:

Before starting the experiment, a 30-minutes period of open perfusion was allowed to wash out the blood from the intervillous space and the villous vascular compartment and to allow recovery of the placental tissue from the ischemic period after delivery. The medium was exchanged on the maternal and fetal side with a volume of 250 ml of new medium in each. A therapeutic dose of erythromycin (2 μg/ml) was applied to the medium in the maternal circuit. Antipyrine (50ug/ml) which is known as a passively diffused substance was used as a reference compound to detect failure of perfusion. After stabilization of the perfusion with medium and antipyrine only, the experiment started and lasted for 240 minutes.

### Samples:

Samples of perfusate were obtained from the maternal and fetal circulations at 0, 15, 30, 60, 120 and 240 minutes by a 5ml syringe connected to a 5cm tube. For biochemical assay, antipyrine detection, 2ml samples of perfusate were centrifuged for five minutes at 4000 Xg and 4ºC. The supernatants were stored at -20ºC until analysis was done. Tissue samples weighing about one gram were cut from placenta before perfusion started (T_0_). Other tissue samples of the same weight were obtained from the area selected for perfusion at the end of the perfusion, from inside the perfusion chamber from both the perfused and unperfused areas. The tissue samples were stored at –20°C. The frozen tissues were homogenized at 4°C with a homogenizer. The homogenate was centrifuged at 4000 X g for ten minutes and stored at -80°C until assayed.

### Analysis:

***Glucose and Lactate:*** Glucose and lactate production were assayed using r X monza clinical analyser (Randox instruments). Amount of glucose consumption was calculated from the difference between initial and final levels in medium of maternal and fetal sides and were normalized to wet tissue weight and perfusion time length.^8^

***HPLC:*** Erythromycin concentration was determined by high-performance chromatography according to the method described by Griessmann.^9^ Instrument pumps Waters 616; detector; Waters PDA 996; Autosampler; Waters 717. The lower limit of quantitation was 50 ng/ml. The transfer rate of antipyrine was determined by high-performance liquid chromatography (HPLC) as described by Challier.^10^ The measurement of hCG, leptin and cytokines concentrations were performed using Bioteck Instrument -- (ELIZA) from –BIOTECK Instrument, Inc. Highland Park.

***Calculation:*** The glucose consumption and lactate production were calculated from difference between initial and final levels in perfuaste on both maternal and fetal side, and normalized to tissue weight and length of perfusion period. The net production of HCG and leptin after four hours was calculated with formula NP = A_Tot_ + T_E_ – T_0_, where A_tot_ means the hormone accumulation in the perfusate, T_E_ the tissue content determined after perfusion and T_0_ the tissue content before initiation of the experiment. Results were normalized to tissue weight and to T_0._

## RESULTS

### Viability of placental tissue:

The viability of the perfused placental lobules is determined by pH (7.2-7.4), fetal to maternal leak less than 4 ml/h and other viability parameters. Table-I shows the characteristics of participant mothers and their newborns, and Table-II shows glucose consumption, lactate production, HCG secretion as evidence of placenta viability and metabolic activity.

**Table-I:** Characteristics of mothers and their newborns (n=6).

Characteristic	Mean ± SD
Mother’s age (years)	28.8 ± 2.9
Body Mass Index (BMI)	29.4 ± 3.2
Gestational age (Weeks)	33+2
Pregnancy complication	None
Used antibiotic during pregnancy	No
Diagnosed with fetal growth restriction	No

*Mode of delivery*	*Spontaneous vaginal*

Baby weight (grams)	3305 ± 209
Placenta weight (grams)	600 ± 52
Cotyledon weight (grams)	15.23 ± 2.61

**Table-II:** Placental viability and metabolic activity (n=6).

	Maternal	Fetal
Glucose consumption (mol/g/min)	0.42 ± 0.04	0.35 ± 0.0.04
Lactate production (mol/g/min)	0.49 ± 0.14	0.31 ± 0.06
HCG (mU/g/min)	40.8 ± 7.3	

### Antipyrine in placental perfusion:

**Fig.1:**
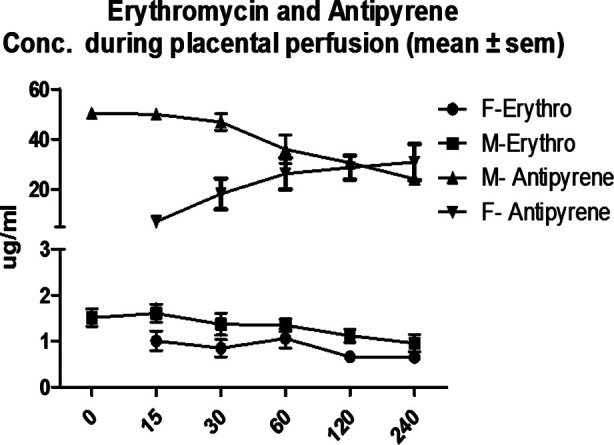
The Trans placental of antipyrine and erythromycin (the data are means of 6 placentas).

The reference antipyrine was detected in the fetal circulation in the first 15 minutes after addition of the drug. At this point the mean antipyrine was 49.90±2.10μg/ml in the maternal perfusate and 7.1±1.56μg/ml in fetal perfusate (Fig.1). The fetal and maternal concentration became similar at 120 minutes. The transfer of antipyrine from maternal to fetal compartment was 98.66%. The differences between perfusion groups were non-significant that indicates the perfusion of placentas is comparable to each other.

**Table:** Data

Time (minutes)	Maternal Erythromycin	Fetal Erythromycin	Maternal Antipyrine	Fetal Antipyrine
0	1.51±1.91		50.30±0.83	
15	1.61±0.19	1.01±0.189	49.90±2.10	7.1±1.56
30	1.37±0.24	0.85±0.19	46.90±3.34	18.20± 6.15
60	1.35±0.13	1.06±0.2017	36.0±5.6	26.30±6.29
120	1.12±0.14	0.66±0.044	30.6±1.6	28.70±4.78
240	0.96±0.19	0.65±0.04	24.20±1.15	30.90±7.24

### Erythomycin in placental perfusion:

**Fig.2:**
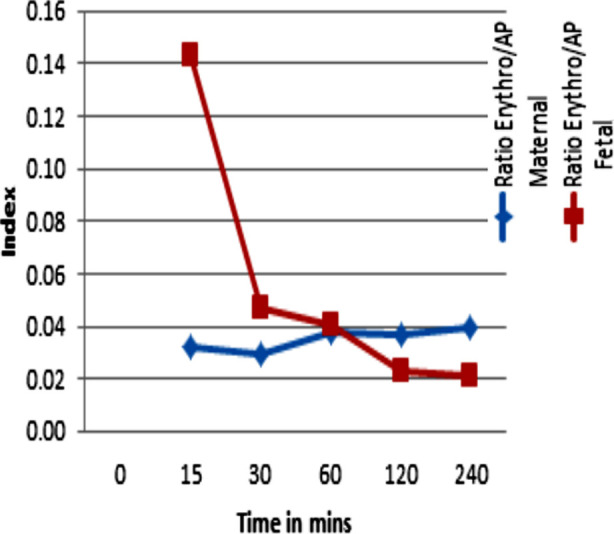
Percentage Transfer Index of Erthromycin / Antipyrine.

After media exchange in both sides, the test compound erythromycin was added to the maternal perfusate. The experimental period of four hours was continued with medium circulation on both maternal and fetal circulation. The concentration of erythromycin decreased in the maternal circuit and increased in the fetal circuit (Fig.2). Table-III shows levels of Interleukin six in maternal perfusate.

**Table-III:** Levels of Interleukin six in maternal perfusate (pg/ml).

Placenta No	120 minutes	240 minutes
1	4	85
2	10	85
3	85	85
4	70	82
5	18	85
6	40	85

## DISCUSSION

In this study we have demonstrated using the dual perfused placental cotyledon model that Erythromycin shows relatively efficient transfer across the human placenta, achieving a fetal transfer ratio of almost 65% after four hours perfusion. Compared to the control, Antipyrine, transplacental transfer of erythromycin was only slightly slower, a result that was not statistically significant. It showed no effect on the production of placental IL-6. These are clinically relevant findings for obstetric practice given the current understanding of placental function at the fetomaternal interface and outcomes in term and preterm birth.^11^,^12^

The macrolides antibiotics have previously been purported to transfer across the placenta at low level, but the clinical effectiveness shown in clinical trials, meta-analysis and guidelines are in concordance with our findings.^2^,^4^,^6^ The latest guideline^6^ is dated 2017 and it was developed according to a standard methodology. The guideline may need updating in the near future. Based on our observations, there is a biological explanation justifying that erythromycin can be used in the treatment of various maternal infections during pregnancy, and that because of the placental transfer they may also impact on neonatal morbidity. With our results the previously held belief that fetus may be protected by placenta from adverse effects of antibiotic exposure is challenged. There is a need for vigilance about the role placental transfer may play in the development of antibiotic resistance

Erythromycin has been widely used in pregnancy for over half a century and the current firm clinical indications with respect to PPROM and GBS both firm up its future as a clinically relevant antibiotic. PPROM and preterm birth are major public health challenges in both high and low income countries, including Pakistan.^6^,^12^-^14^ Erythromycin following PPROM was associated with a statistically significant reduction in chorioamnionitis, a reduction in the numbers of babies born within 48 hours and seven days, a significantly reduced in neonatal infection reduction in the number of babies with an abnormal cerebral ultrasound scan prior to discharge from hospital.^4^ There is no current serious concern about teratogenicity with macrolides.

There are some recent issues of clinical importance. It should be kept in mind that PPROM can change vaginal microbial composition, and Erythromycin therapy in addition to having a beneficial effect on perinatal outcomes may damage healthy microbiome.^15^-^17^ Moreover, macrolide antibiotics are effective agents that can control intrauterine infection, but macrolide resistance has been documented in Ureaplasma, a known intra-amniotic infection associated with pre-term birth.^18^-^21^ Interestingly, erythromycin prophylaxis is also beneficial in the treatment of feeding intolerance in preterm low birth weight infants.^22^

### Limitation of the study:

The main limitation is that the ex vivo technique is a complex system and expensive and always there were uncontrolled conditions that led to the discontinuation of the experiment and also the availability of the intact placenta. Due to a lack of data regarding placenta transport of antibiotics, our findings may support clinicians’ use of erythromycin in the obstetric field. The manuscript submission was delayed after the completion of the research work, however, the findings are relevant to the current practice. To our knowledge no similar studies have been done since we conducted our study and no newer methods have emerged since 2013.

## CONCLUSION

Our study suggests that erythromycin prescribed to pregnant women transfers across the placenta and affects inflammatory markers impacting perinatal morbidity. Our findings also point toward the importance of further studies concerning neonatal adverse effects and the development of antibiotic resistance.

### Authors’ Contribution:

**HME** and **NO** conceived and designed the study, collected the data, performed analysis, wrote the manuscript and both are responsible and accountable for the accuracy or integrity of the work.

All authors did review and gave final approval of the manuscript.
